# Hybrid cardiovascular sourced extracellular matrix scaffolds as possible platforms for vascular tissue engineering

**DOI:** 10.1002/jbm.b.34444

**Published:** 2019-08-01

**Authors:** James A. Reid, Anthony Callanan

**Affiliations:** ^1^ Institute for Bioengineering, School of Engineering The University of Edinburgh Edinburgh UK

**Keywords:** electrospinning, endothelial cells, extracellular matrix, polycaprolactone, scaffold

## Abstract

The aim when designing a scaffold is to provide a supportive microenvironment for the native cells, which is generally achieved by structurally and biochemically imitating the native tissue. Decellularized extracellular matrix (ECM) possesses the mechanical and biochemical cues designed to promote native cell survival. However, when decellularized and reprocessed, the ECM loses its cell supporting mechanical integrity and architecture. Herein, we propose dissolving the ECM into a polymer/solvent solution and electrospinning it into a fibrous sheet, thus harnessing the biochemical cues from the ECM and the mechanical integrity of the polymer. Bovine aorta and myocardium were selected as ECM sources. Decellularization was achieved using sodium dodecyl sulfate (SDS), and the ECM was combined with polycaprolactone and hexafluoro‐2‐propanol for electrospinning. The scaffolds were seeded with human umbilical vein endothelial cells (HUVECs). The study found that the inclusion of aorta ECM increased the scaffold's wettability and subsequently lead to increased HUVEC adherence and proliferation. Interestingly, the inclusion of myocardium ECM had no effect on wettability or cell viability. Furthermore, gene expression and mechanical changes were noted with the addition of ECM. The results from this study show the vast potential of electrospun ECM/polymer bioscaffolds and their use in tissue engineering.

**Abbreviations**
ANOVA: analysis of variance
CD31/PECAM1: platelet endothelial cell adhesion molecule
DAPI: 4′,6‐diamidino‐2‐phenylindole
ECM: extracellular matrix
EDTA: ethylenediaminetetraacetic acid
FBS: fetal bovine serum
FTIR: Fourier transform infrared spectroscopy
GAPDH: glyceraldehyde 3‐phosphate dehydrogenase
HCL: hydrochloric acid
HFIP: hexafluoro‐2‐propanol
HUVEC: human umbilical vein endothelial cell
MMP1: matrix metalloproteinase‐1
MMP2: matrix metalloproteinase‐2
PBS: phosphate buffer saline
PCL: polycaprolactone
RT‐qPCR: quantitative reverse transcription polymerase chain reaction
SDS: sodium dodecyl sulfate
SEM: scanning electron microscope
VEGF: vascular endothelial growth factor

## 1 INTRODUCTION

In 2015, cardiovascular disease killed around 630,000 people in the United States, accounting for 23.4% of all deaths (Heron, 2017). Approximately 80% of those deaths fell in the category of over 65 year olds (Heron, 2017). One of the major reasons for the increase in people requiring sophisticated and complex cardiac interventions is the ever increasing average life expectancy across the world's population (Blanche et al., 2001). For example, it was found that between 1999 and 2010, in excess of 83% of over 60 year olds in the USA were found to have hypertension or prehypertension symptoms (Guo, He, Zhang, & Walton, 2012). Alongside this, over 7.6 million premature deaths were noted globally in 2001 due to cardiovascular disease, with this number rising (Lawes, Vander, & Rodgers, 2008; Makridakis & DiNicolantonio, 2014). With such a high prevalence in the elderly population and a rapidly increasing global ageing population, it is of no surprise that the demand for vascular treatments is increasing (United Nations, 2015).

There are currently a large variety of vascular tissue engineering strategies utilized in the treatment of arterial disease (Ravi & Chaikof, 2010). These include scaffold materials that mimic the native extracellular matrix (ECM), promote ECM production, reduce inflammation and thrombogenicity, and stimulate neovascularization and angiogenesis (Jiang, Akgun, Lam, Ameer, & Wertheim, 2015; Ravi & Chaikof, 2010). Materials used in vascular tissue engineering include decellularized extracellular matrices, biopolymers, bioabsorbable polymers, and collagen (Gilbert, Sellaro, & Badylak, 2006; Hong et al., 2008; Hutmacher, 2000; Jiang et al., 2015; Lu, Lin, Kim, et al., 2013; Masoumi et al., 2014; Reid & Callanan, 2019; Wu, Liu, Cui, Qu, & Chen, 2007). Electrospinning is a widely used technique that mimics the nanoscale and microscale structure of tissues and has been utilized in a large number of tissue engineering avenues for various organs (Burton & Callanan, 2018; Burton, Corcoran, & Callanan, 2017; Dettin et al., 2015; Gao et al., 2017; Garrigues, Little, Sanches‐Adams, et al., 2014; Grant, Hay, & Callanan, 2017; Han, Gerstenhaber, Lazarovici, & Lelkes, 2013; Hong et al., 2008; Kumbar, Nukavarapu, James, Nair, & Laurencin, 2008; Masoumi et al., 2014; Sundaramurthi, Krishnan, & Sethuraman, 2014). Furthermore, while polymers can control architecture, they lack some of the unique biomolecular cues found in native tissue, which is why, in recent years, there has been an increase in demand for decellularized ECM (Kasimir et al., 2006; Sanchez, Fernandez‐Santos, Costanza, et al., 2015; Tapias & Ott, 2014; Weymann, Patil, Sabashnikov, et al., 2014). The ECM is the pivotal factor in the intracellular microenvironment, thus it plays a major role in maintaining and regulating tissue function (Kang, Kim, Khademhosseini, & Yang, 2011). Therefore, incorporating components of the ECM into a scaffold to more closely mimic the native environment is an effective way to promote tissue regeneration (Bhowmick et al., 2017; Choi et al., 2010). While there have been huge advances in the treatment of cardiovascular disease, there is still a large demand to increase patency and surgical outcome.

Hybrid scaffolds that incorporate ECM with a polymer into a spun fiber have shown improved cellular performance when using meniscus and cartilage (Gao et al., 2017; Garrigues et al., 2014). Other methods of manufacturing scaffolds/constructs using vascular ECM and polymers have ranged from repopulating aortic valves to vascular grafts in the cardiovascular realm (Schoen, Avrahami, Baruch, et al., 2017). Interestingly, studies focusing on vascular ECM have shown improvements in native cell repopulation and mechanical properties when combining the ECM with a polymer (Hong et al., 2008; Jahnavi, Kumary, Bhuvaneshwar, Natarajan, & Verma, 2015; Jiang et al., 2015; Wu et al., 2007). Furthermore, electrospinning was successfully utilized to generate these hybrid structures that showed increased mechanical strength and improved cellular repopulation (Hong et al., 2008; Jahnavi et al., 2015; Wu et al., 2007). These previous studies combining vascular ECM and polymers to create hybrid scaffolds show promising results, with improvements in cellular performance noted in most cases.

Current treatments are not keeping up with the increased demand for surgery and transplantation (Blanche et al., 2001; Kilic, Emani, Sai‐Sudhakar, Higgins, & Whitson, 2014). Herein, we propose incorporating the native vascular ECM into the electrospun polymer fibers via solubilization of the ECM. By combining heart and aorta ECM with polycaprolactone (PCL), the aim is to manufacture an improved platform for the attachment and growth of endothelial cells, with the aim of improving vascular regeneration.

## 2 MATERIALS AND METHODS

### 2.1 ECM production

Bovine heart and aorta were harvested from a 2‐year‐old female. Samples were frozen within 4 hr of harvesting at −80°C. Heart tissue was sliced up into 5‐mm‐thick slices using a meat slicer. The aorta was opened up and flattened out and subsequently had 40‐mm‐circular samples punched out. This was repeated for the heart slices. Slices were cleaned with ethanol and placed in water for 30 min to remove excess blood. Samples were then cut up into 2 mm × 2 mm square pieces to increase surface area for decellularization. Pieces were perfused with 0.5% w/v sodium dodecyl sulfate (SDS) in deionized water for 36 hr at 170 mL/min. Samples were then perfused with 10 L of deionised water at 170 mL/min. Decellularized samples were then frozen at −80°C before being lyophilized in a FreeZone® 4.5 freeze‐drier (Labconco®). Dry ECM was then crushed up into ~0.5 mm pieces before being milled in a planetary ball mill PM 100 (Retsch). ECM powder was then collected in water, frozen at −80°C, and lyophilized leaving behind ECM powder. Powder was stored at 4°C.

### 2.2 Histology

Native and decellularized samples of bovine heart and aorta were fixed in 10% formalin for 24 hr. Samples were then dehydrated in a series of ethanol washes starting at 50% concentration and ending at 100% concentration. Samples were washed in xylene before being embedded in paraffin wax and then stored at 4°C. All samples were then trimmed to 5 μm thicknesses and mounted onto glass slides for haematoxylin and eosin (H&E) staining and Picrosirius Red staining. Samples were cleared in xylene before staining and mounted in Distyrene Plasticizer Xylene (DPX) after staining.

#### 2.2.1 Haematoxylin and eosin staining

Samples were cleared of paraffin before undergoing rehydration in a series of ethanol and H_2_O washes. Samples were stained in haematoxylin for 6 min and Eosin for 90 s, leading to a vivid pink color for the eosinophilic substances (cell cytoplasm and ECM proteins) and a dark blue/black for the nuclear substances (cell nucleus).

#### 2.2.2 Picrosirius red staining

Samples were cleared of paraffin before undergoing rehydration in a series of ethanol and H_2_O washes. Samples were stained in 0.1% Sirius Red for 1 hr, leading to a vivid red color for the collagenous substances (ECM) and a dull yellow color for the cellular substances (cell cytoplasm and cell nucleus) (Velidandla, Gaikwad, Ealla, et al., 2014).

### 2.3 Scaffold manufacture (electrospinning)

**Figure 1:**
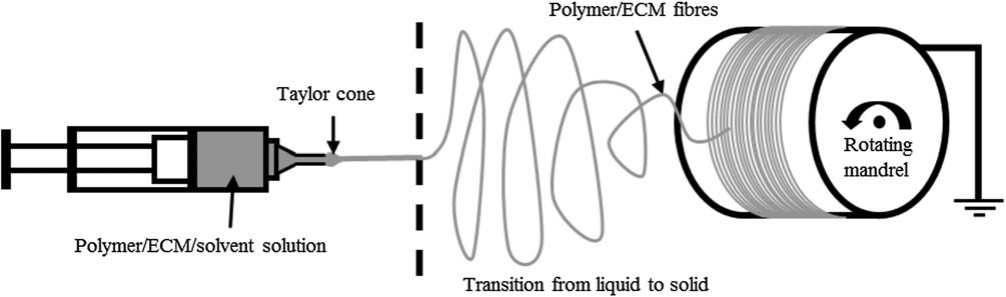
Electrospinning setup. The polymer/solvent solution is drawn across from the Taylor cone formed at the needle tip to the charged rotating mandrel. As the solution is drawn across, it transitions from a liquid into a solid fiber that attaches itself to the rotating mandrel

Powdered heart or aorta ECM was dissolved at 0.25% w/v in hexafluoroisopropanol (HFIP). PCL was then dissolved at 8% w/v into the HFIP/ECM solution overnight with agitation. The solutions were placed into a 20 mL syringe and pumped using a syringe pump EP‐H11 (Harvard Apparatus) into the EC‐DIG electrospinning system (IME Technologies). PCL/ECM scaffolds were electrospun using the following parameters: 0.4 mm needle bore, 0.8 mL/hr volume flow rate (6 mL in total), 12 cm mandrel: needle distance, +14 kV positive charge, −4 kV negative charge, and 250 revolutions per minute mandrel rotational speed. These parameters have been shown to create uniform randomly aligned small fibers of PCL (Burton et al., 2017). The mandrel was coated with aluminum foil for collection. Once collected, the electrospun sheet was left to dry for 24 hr in the hood before being stored at 4°C. Scaffolds were punched out using a 10 mm punch and left in ethanol for 30 min to sterilize and assist with removal from the aluminum foil. The 0.25% ECM scaffolds had final PCL:ECM ratios of 96.97:3.03 (approximated to 97:3 for simplicity). This ratio was deduced from the remnant PCL:ECM ratio left over once the solvent had left the solution, which was 8% w/v PCL:0.25% w/v ECM. The electrospinning process is shown in Figure 1.

### 2.4 Scanning electron microscopy

Both unseeded and seeded scaffolds were visualized at day 0 and day 10 using a Hitachi S4700 fueled emission scanning electron microscope (SEM, Hitachi) with a 5 kV accelerating voltage and a working distance of 12 mm. Briefly, the day 10 seeded scaffolds (*n* = 2) were fixed in 4% v/v glutaraldehyde overnight, before being stored in phosphate buffer saline (PBS) at 4°C for 3 days. Scaffolds were then incubated in 0.1% v/v osmium for 30 min, followed by four rinses in DiH_2_O. Samples were then dehydrated in ethanol using 30 s intervals of increasing ethanol concentration from 30 to 100% v/v to achieve critical point drying. Scaffolds were then placed in HDMS for 1 min. HDMS was then replaced and left to evaporate overnight in a fume cupboard. Prior to imaging, all scaffolds were sputter coated using an Emscope SC500A splutter coater using gold–palladium (60:40).

The process of glutaraldehyde fixation followed by Osmium tetroxide staining allows for clear viewing of the cellular membrane and ECM components on the scaffold. Glutaraldehyde fixation causes rapid crosslinking of the proteins within the cells (Hopwood, 1972). Osmium tetroxide is then used as a lipid stain by embedding a heavy metal directly into the cell membrane, thus creating a high electron scattering rate allowing for clear visualization of the cell membrane (Thiery, Bernier, & Bergeron, 1995).

### 2.5 Mechanical testing

An Instron 3,367 tensile testing machine (Instron, UK) with a 50 N load cell was used to test scaffolds in tension. Young's modulus of the scaffolds was assessed on unseeded scaffolds and on scaffolds after 10 days of culture. Briefly, 20 mm × 4 mm strips of scaffold were cut out for mechanical testing. These samples had a gauge length of 14 mm and were stretched at 10 mm/min until failure or 200% strain was achieved. The incremental Young's modulus was deduced by using the formula:

$$E=\frac{FL_{0}}{A\Delta L}$$

where *E* is Young's modulus, *F* is the applied force, *A* is the cross sectional area, *ΔL* is change in length, and *L*_0_ is the original length. Data were analyzed using a previously described analysis method. Briefly, incremental Young's moduli are calculated to show how stiffness changes with increasing strain (Burton et al., 2017; Burton & Callanan, 2018; Callanan, Davis, McGloughlin, Walsh, 2014a; Callanan, Davis, Walsh, & McGloughlin, 2012; Munir, Larsen, & Callanan, 2018; Munir, McDonald, & Callanan, 2019; Santoro et al., 2018) (*N* = 5 independent replicates).

### 2.6 Contact angle measurement

**Figure 2:**
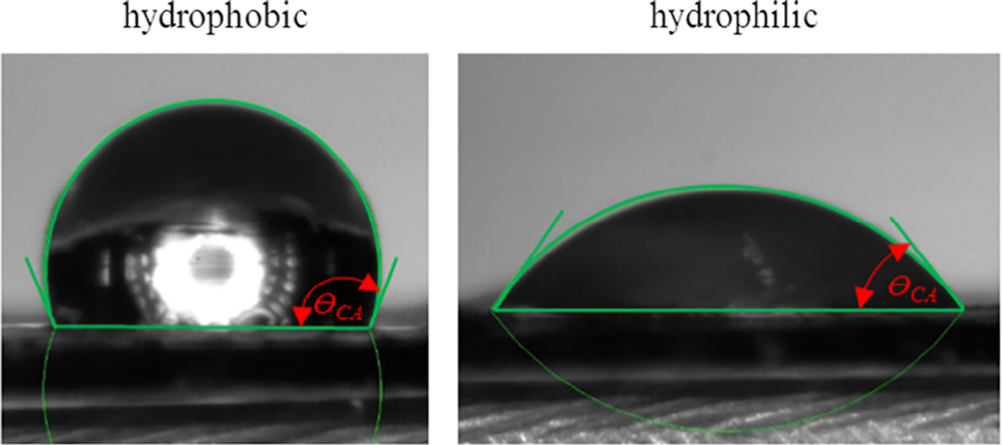
Representative images showing the LBADSA plugin on ImageJ measuring contact angle on a hydrophobic (>90° contact angle) and hydrophilic (<90° contact angle) sample (*Θ*_CA_ = contact angle)

Samples were measured using dry scaffolds. Briefly, a single 5‐μL droplet of water was placed on the scaffold, and images were captured at 5 Hz using a DMK 41AU02 monochrome 1,280 × 960 camera. Analysis was done using ImageJ software with a previously developed plugin: LBADSA (Stalder et al., 2010), as seen in Figure 2 (*N* = 5).

### 2.7 Fourier transform infrared spectroscopy

Fourier transform infrared spectroscopy (FTIR) was used to confirm the successful inclusion of vascular ECMs into the electrospun PCL fibers. Samples were cut into 6‐mm‐diameter scaffolds. All spectra were obtained using a Nicolet™ iS™10 spectrometer with a Smart™ iTX diamond attenuated total reflection detector (all from Thermo Fisher Scientific). Spectra were acquired between 4,000 and 400 cm^−1^ with a resolution of 1 cm^−1^ using OMNIC™ Spectra software (Thermo Fisher Scientific) (*N* = 5 independent replicates).

### 2.8 Cell growth

Human umbilical vein endothelial cells (HUVECs) from an infant male Caucasian donor were obtained cryopreserved at passage 1 (Pro‐moCell GmbH) and expanded to passage 7 in a 5% CO_2_/37°C atmosphere. HUVECs were expanded using MCBD 131 medium (Life Technologies) supplemented with 5% v/v fetal bovine serum (FBS; ThermoFisher Scientific); 1% v/v L‐glutamine; 1% v/v penicillin/streptomycin (Life Technologies); 1 mg/L hydrocortisone; 50 mg/L ascorbic acid (Sigma); 2 μg/L fibroblast growth factor (PeproTech); 10 μg/L epidermal growth factor (PeproTech); 2 μg/L insulin‐like growth factor (PeproTech); and 1 μg/L vascular endothelial growth factor (PeproTech) (Callanan, Davis, McGloughlin, Walsh, 2014b; Carroll, McGloughlin, O'Keeffe, et al., 2009; Davis et al., 2014).

### 2.9 Cell seeding and culture

Scaffolds were punched out using a 10 mm punch and left in 70% v/v ethanol for 30 min before being seeded. Scaffolds were plated into a 48 well plate and were then presoaked in serum free MCDB 131 medium (same cocktail as cell growth medium minus FBS) overnight to increase their hydrophilicity. Cells were seeded at 35,000 cells per scaffold. Briefly, a 35,000 cell suspension in 20 μL of medium was dropped onto the middle of the scaffold and left for 30 min, before an additional 30 μL of medium was added to ensure that the cells did not dry up. After another 30 min, 450 μL of medium was added, increasing the total volume of media to 500 μL. Seeded scaffolds were fed every 2 days using the same MCBD 131 medium cocktail as previously described.

Scaffolds for mechanical testing were cut out at 20 mm × 4 mm and seeded at the same seeding density as the 1‐mm‐diameter scaffolds.

### 2.10 CellTiter‐blue® cell viability assay

The assay was performed at 1, 5, and 10 days as the manufacturer's instructions (Promega). Briefly, scaffolds were moved into new media in a fresh 48‐well plate to prevent reading the activity of cells bound to the tissue culture plastic. Measurements were made with a Modulus™ II microplate reader at Excitation 525 nm and Emission 580–640 nm (*N* = 4 independent replicates).

### 2.11 DNA quantification

Cell seeded scaffolds cultured for 1, 5, and 10 days were frozen and lyophilized before being incubated in a papain digest solution of 2.5 U papain, 5 mM cysteine HCL, and 5 mM EDTA in DNA free water (all reagents from Sigma‐Aldrich, UK) at 60°C for 24 hr and periodically mixed using a vortexer. Total DNA content of the samples was calculated using a Quant‐iT™PicoGreen® assay kit (ThermoFisher, UK) as per the manufacturers' instructions. Fluorescence was read using a Modulus™ II microplate reader at Excitation 490 nm and Emission 510–570 nm (*N* = 4 independent replicates).

### 2.12 Cell imaging

Scaffolds were washed thrice in PBS and fixed using 500 μL of 4% v/v formalin solution in PBS for 24 hr, then washed again three times in PBS. Permeabilization achieved by placing scaffolds in 0.2% v/v TritonX‐100 solution in PBS for 5 min, followed by three PBS washes.

Scaffolds were stained with 0.1 mL of 0.1 μL 1000X Phalloidin‐iFluor™514 conjugate (AAT Bioquest, Stratech) in 0.1 mL PBS with 1% v/v bovine serum albumin for 60 min. Scaffolds were washed thrice in PBS for 10 min. Scaffolds were then stained with 0.1 mL of 300 nM 4′,6‐diamidino‐2‐phenylindole (DAPI) (Sigma‐Aldrich, UK) in PBS for 10 min followed by three 10 min PBS washes.

All scaffolds were imaged using a Coherent Anti‐Stokes Raman scattering microscope.

### 2.13 Real‐time RT‐qPCR

RNA was extracted from the scaffolds using standard Tri‐Reagent (Invitrogen, ThermoFisher) methods and purified using Qiagen's RNeasy spin column system. Real‐time polymerase chain reaction was performed using a LightCycler® 480 Instrument II (Roche Life Science) and Sensifast™ SYBR® High‐ROX system (Bioline). Forward and reverse sequences were designed with Sigma‐Aldrich and are displayed in Table 1 (Callanan et al., 2014b; Fischer et al., 2009). Relative quantification of Reverse transcription polymerase chain reaction (RT‐PCR) results was carried out using the 2^−ΔΔ*ct*^ method (Livak & Schmittgen, 2001). Gene expression levels were expressed relative to Glyceraldehyde 3‐phosphate dehydrogenase (GAPDH) (housekeeping gene) and normalized to expression on the day 1 PCL control.

**Table 1:** The sequences for forward and reverse gene specific primers used in RT‐PCR amplification

Gene	Primer	Sequence	Sequence length	Reference
Glyceraldehyde 3‐phosphate dehydrogenase	GAPDH (forward)	GTCTCCTCTGACTTCAACAG	20	(Callanan et al. 2014b)
	GAPDH (reverse)	GTTGTCATACCAGGAAATGAG	21	
Matrix metalloproteinase‐1	MMP1 (forward)	CGGTTTTTCAAAGGGAATAAGTACT	25	(Callanan et al. 2014b)
	MMP1 (reverse)	TCAGAAAGAGCAGCATCGATATG	23	
Matrix metalloproteinase‐2	MMP2 (forward)	CGCTCAGATCCGTGGTGAG	19	(Callanan et al. 2014b)
	MMP2 (reverse)	TGTCACGTGGCGTCACAGT	19	
Vascular endothelial growth factor	VEGF (forward)	AGACCAAAGAAAGATAGAGCAAGACAAG	28	(Callanan et al. 2014b)
	VEGF (reverse)	GGCAGCGTGGTTTCTGTATCG	21	
Platelet endothelial cell adhesion molecule	CD31 (forward)	ACTGGACAAGAAAGAGGCCATCCA	24	(Fischer et al. 2009)
	CD31 (reverse)	TCCTTCTGGATGGTGAAGTTGGCT	24	

### 2.14 Statistical analysis

Data were expressed as mean ± 1 *SD*, unless stated otherwise. Statistical analysis was performed using one‐way analysis of variance (ANOVA) with post hoc Fisher test for DNA content, CellTiter‐Blue®, and real‐time qPCR, unless stated otherwise. One‐way ANOVA with post hoc Fisher's test has been widely used in literature when comparing a large range of variable sizes (Bédouin, Gordin, Pellen‐Mussi, et al., 2018; Nayak & Brenner, 2002; Vogels et al., 2017; Woods & Gratzer, 2005).

## 3 RESULTS

### 3.1 Decellularization and scaffold manufacture

**Figure 3:**
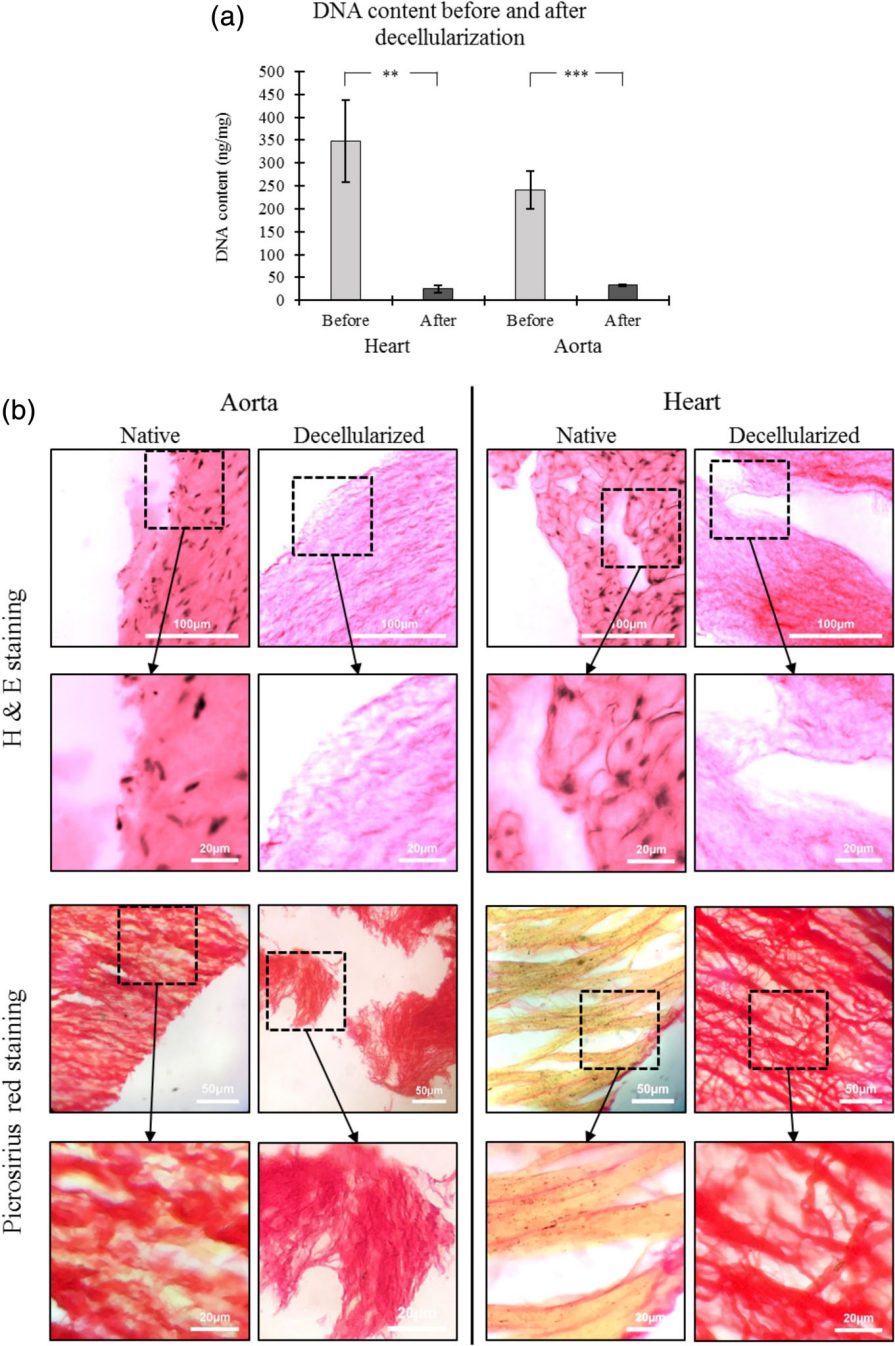
(a) DNA content of aorta and heart before and after decellularizing treatment. Data shown as mean ± 1 *SD*, student 2 sample *t*‐test performed (**p < .01, ***p < .001, and *n* = 4). (b) H&E staining and Picrosirius red staining of the aorta and heart tissue before and after decellularization. H&E staining shows a vivid pink color for the eosinophilic substances (cell cytoplasm and ECM proteins) and a dark blue/black color for the nuclear substances (cell nucleus). Picrosirius red staining shows a vivid red color for the collagenous substances (ECM) and a dull yellow color for the cellular substances (cell cytoplasm and cell nucleus). ECM, extracellular matrix; H&E, haematoxylin and eosin

**Figure 4:**
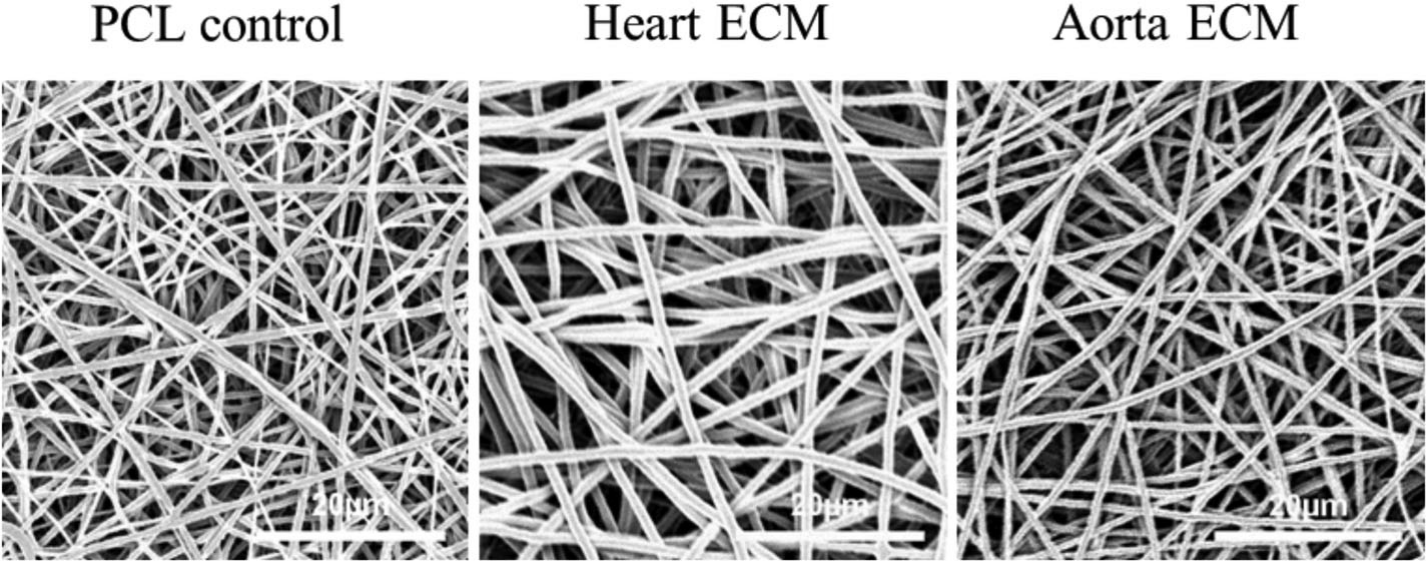
Scanning electron microscopy images of the three electrospun sheets. All three scaffolds appear to have a smooth fiber structure and show relatively uniform fiber diameter throughout (fiber diameters of 0.97 ± 0.19, 1.20 ± 0.09, and 0.94 ± 0.12 μm, respectively, for the PCL, heart ECM, and aorta ECM scaffolds). ECM, extracellular matrix; PCL, polycaprolactone

Bovine heart and aorta ECM were successfully decellularized using 0.5% w/v SDS as seen in Figure 3a. H&E staining showed that the cells were successfully removed from both tissues (see Figure 3b). Furthermore, Picrosirius red staining showed that the collagenous composition of the ECM was maintained after decellularization (Figure 3b). Electrospun fibrous sheets (as seen in Figure 4) were successfully fabricated using the previously described parameters (Burton et al., 2017). The fibers within each scaffold are fairly uniform in size (fiber diameters of 0.97 ± 0.19, 1.20 ± 0.09, and 0.94 ± 0.12 μm, respectively, for the PCL, heart ECM, and aorta ECM scaffolds) and show a randomness in their orientation. Furthermore, the fibers appear to be smooth in structure.

### 3.2 Fourier Transform Infrared Spectroscopy

**Figure 5:**
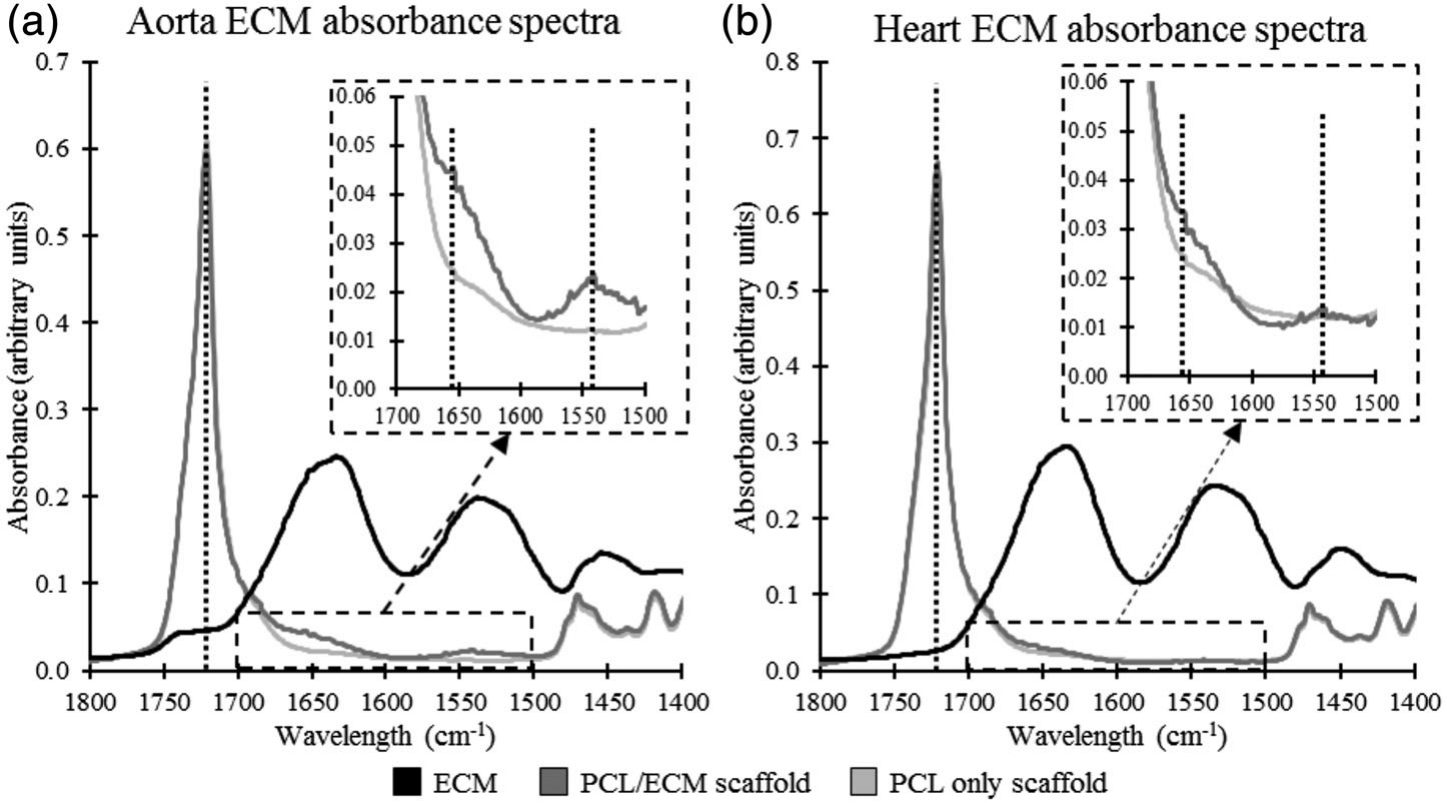
Representative FTIR absorbance spectra for (a) aorta ECM scaffolds and (b) heart ECM scaffolds. ECM, extracellular matrix; FTIR, Fourier transform infrared spectroscopy

The FTIR results showed that the bovine heart and aorta ECMs were incorporated into the electrospun PCL fibers. Spectra were taken for PCL fibers, ECM/PCL fibers, and ECM alone. Characteristic peaks from both the PCL and ECM were found in the ECM/PCL fibers demonstrating that the electrospinning process integrated the decellularized ECM into the PCL fiber. Figure 5 shows peaks in absorbance at 1720, 1654, and 1,541 cm^−1^ for both heart and aorta ECM (See Supplementary Figure S1 in the appendix for overlay graphs of all five samples for each group). These peaks can be attributed to the stretching of the carbonyl group in PCL, the amide I bond in the ECM, and the amide II bond in the ECM, respectively (Camacho, West, Torzilli, & Mendelsohn, 2001; Elzein, Nasser‐Eddine, Delaite, Bistac, & Dumas, 2004; Garidel & Schott, 2006). These values are shown in Table 2. Furthermore, differences in absorbance values between PCL only scaffolds and the PCL/ECM scaffolds were quantified at each wavelength to show that the differences were observed due to the inclusion of ECM. Briefly, increases of 84.3 ± 8.0% (*p* < .01) and 109.2 ± 18.6% (*p* < .01) were seen at 1654 and 1,541 cm^−1^, respectively, for the aorta ECM, with a decrease of 19.4 ± 15.5% (*p* < .05) at 1720 cm^−1^. The heart ECM showed increases of 35.2 ± 6.7% (*p* < .01) and 26.8 ± 10.7% and a decrease of 10.5 ± 15.2% for the three wavelengths. These values are displayed in Table 3.

**Table 2:** Absorbance values for each peak

Wavelength of peak (cm^−1^)	Absorbance values		
	PCL	Aorta ECM	Heart ECM
1,720	0.774 ± 0.110	0.624 ± 0.040	0.693 ± 0.039
1,654	0.025 ± 0.001	0.046 ± 0.004	0.034 ± 0.002
1,541	0.011 ± 0.001	0.023 ± 0.004	0.014 ± 0.001

Abbreviations: ECM, extracellular matrix; PCL, polycaprolactone.

**Table 3:** Wavelength of peaks noted in FTIR spectra

Wavelength of peak (cm^−1^)	Assignment	Caused by	Change in absorbance between PCL fiber and ECM/PCL fiber	
			Aorta ECM	Heart ECM
1,720	Carbonyl group stretching	PCL	− 19.4 ± 15.5%	− 10.5 ± 15.2%
1,654	Amide I bond	ECM (collagen and elastin)	+ 84.3 ± 8.0%	+ 35.2 ± 6.7%
1,541	Amide II bond	ECM (collagen and elastin)	+ 109.2 ± 18.6%	+ 26.8 ± 10.7%

Abbreviations: ECM, extracellular matrix; PCL, polycaprolactone.

### 3.3 Mechanical testing

**Figure 6:**
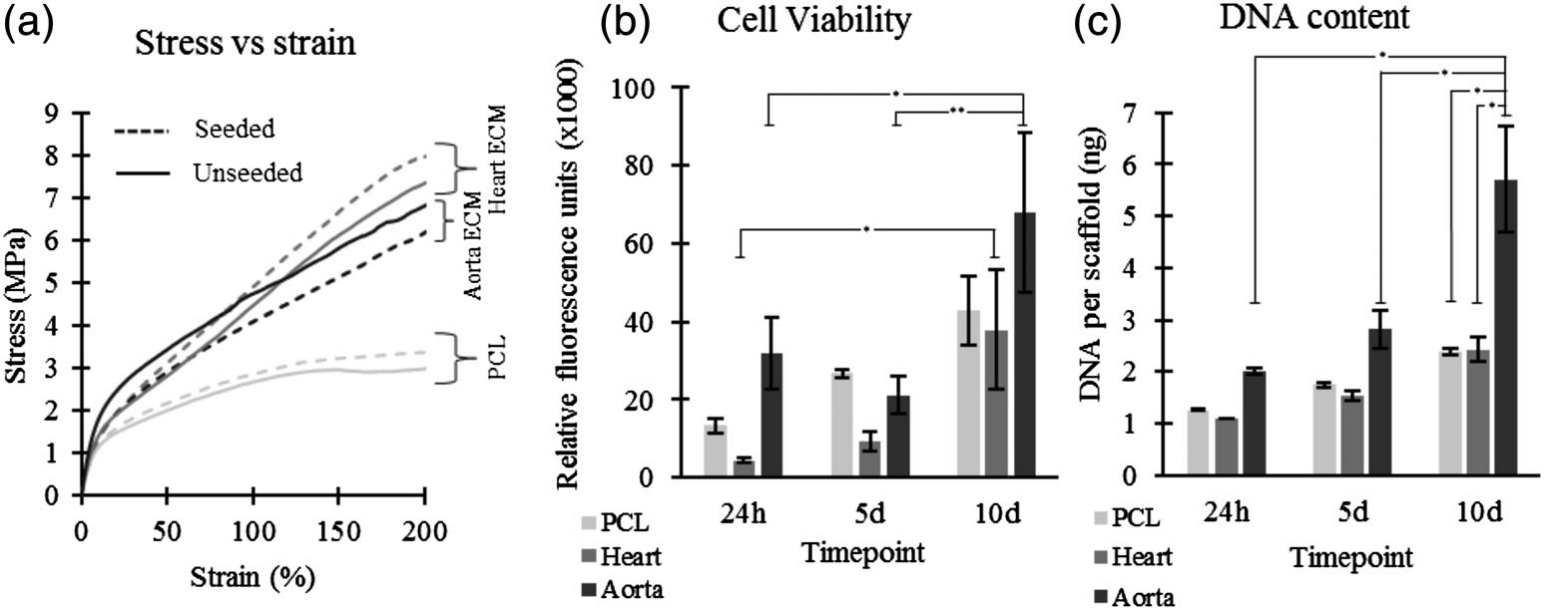
(a) Representative stress versus strain graphs of each scaffold—Unseeded and after 10 days of HUVEC culture. (b) Cell viability of scaffolds analyzed using CellTitre blue® fluorescence assay. A statistically significant increase in cell viability was seen in the heart ECM and aorta ECM scaffolds between day 1 and day 10. (c) DNA content of scaffolds assessed using a Picogreen assay. The aorta ECM showed the largest increase in DNA content between day 1 and day 10 and showed a significantly higher DNA content than the PCL scaffold and heart ECM scaffold at day 10. *n* = 4, error bars = 1 *SD*, one‐way ANOVA with Fishers' post hoc performed (*p < .05 and **p < .01). ANOVA, analysis of variance; ECM, extracellular matrix; HUVEC, human umbilical vein endothelial cell

Mechanical testing performed using an Instron tensile tester showed no statistically significant difference between cell seeded and unseeded scaffolds for each scaffold at all the strain bands. However, the general trend for the PCL scaffold and the heart ECM scaffold is a slightly higher Young's modulus in the seeded scaffolds then the unseeded scaffold, as seen in Table 4. However, as previously mentioned, this difference is not significant. The only statistical significance is in the aorta ECM scaffold for the 3–4% strain band, where the seeded scaffold was significantly lower than the unseeded scaffold (*p* < .05). The general trend noted is that the two ECM scaffolds had consistently higher Young's moduli at all strain brackets compared with the PCL control. This was significant at the 0–1% strain band for both scaffolds where the heart ECM was 23.2% higher and aorta ECM 28.4% higher (*p* < .05); and the 1–2% strain band for aorta ECM which was 31.0% higher (*p* < .05). As expected, Young's modulus drops at each subsequent strain bracket for all the scaffold (with the exception of unseeded aorta ECM scaffold between the 0–1% and 1–2% strain brackets). Representative stress–strain graphs for each scaffold are displayed in Figure 6a.

**Table 4:** Mechanical properties of the three scaffolds

	Scaffold type					
	PCL		Heart ECM		Aorta ECM	
	Unseeded	Seeded	Unseeded	Seeded	Unseeded	Seeded
Young's modulus at % strain (MPa)						
0–1	17.05 ± 1.81	19.48 ± 2.14	21.00 ± 1.87	22.58 ± 2.13	21.90 ± 2.93	21.60 ± 1.91
1–2	16.80 ± 1.27	18.10 ± 2.21	20.87 ± 2.60	20.68 ± 2.37	22.00 ± 3.11	18.76 ± 1.95
2–3	15.03 ± 1.89	15.25 ± 2.47	16.27 ± 2.22	18.18 ± 1.62	18.04 ± 2.29	15.82 ± 1.12
3–4	13.93 ± 1.53	14.55 ± 3.00	15.96 ± 1.69	15.84 ± 1.60	17.34 ± 1.95	14.30 ± 0.85
4–5	12.45 ± 1.75	13.08 ± 2.80	12.64 ± 1.40	14.16 ± 1.18	14.90 ± 1.76	12.92 ± 0.64
0–5^a^	15.05 ± 1.54	16.09 ± 2.49	17.35 ± 1.83	18.29 ± 1.72	18.84 ± 2.28	16.68 ± 1.26
Contact angle after 0.2 s (degree)	114.4 ± 8.9		122.5 ± 3.5		89.6 ± 37.7	
Contact angle after 1 s (degree)	103.6 ± 19.6		118.8 ± 10.7		72.8 ± 43.9	
Contact angle after 5 s (degree)	98.7 ± 25.2		106.3 ± 27.2		68.8 ± 43.6	
Percentage drop in contact angle between 0.2 and 1 s	9.44%		3.02%		18.75%	
Percentage drop in contact angle between 0.2 and 5 s	13.72%		13.22%		23.21%	
Fiber diameter (μm)	0.97 ± 0.19		1.20 ± 0.09		0.94 ± 0.12	

*Note*: Values displayed ± 1 *SD*.

Abbreviations: ECM, extracellular matrix; PCL, polycaprolactone.

a Young's modulus across the entire strain range from 0 to 5% strain.

### 3.4 Contact angle

The addition of aorta ECM had the effect of increasing the hydrophilicity of the scaffold compared with the conventional PCL scaffold and heart ECM scaffold, as seen in Table 4. These results were significant when comparing the aorta ECM scaffold with the heart ECM scaffold—26.9% lower (*p* < .05), but not when comparing either with the PCL scaffold. Furthermore, the percentage drop in contact angle between 0.2 s postcontact and 5 s postcontact was higher for the aorta ECM scaffold than the two others.

### 3.5 Cell viability and DNA quanitification

Cell viability was assessed using a CellTitre‐Blue® fluorescence assay. Results (Figure 6b) showed that after 10 days, the aorta ECM scaffold and the heart ECM scaffold both showed a significant increase in cell viability compared with viability after 1 day (*p* < .05). The aorta ECM scaffold also showed a significant increase compared with its viability after 5 days (*p* < .01). On the other hand, the PCL scaffold showed no significant increase in cell viability between all three time points, although an upward trend in cell viability is noted. After 10 days, the aorta ECM scaffold had a 40% higher cell viability than the heart ECM and the PCL scaffold. However, this increase was not found to be significant.

DNA quantification was performed using a Picogreen assay. The DNA content (Figure 6c) of the aorta ECM scaffolds at 10 days is significantly different from the aorta ECM scaffold at day 1 (a 275% increase). Alongside this, the day 10 aorta ECM scaffold was also significantly higher by approximately 240% than the heart ECM and PCL scaffolds (*p* < .05). The trend noted across the three time points is an increase in DNA content for all three scaffolds, but a larger increase in the aorta ECM scaffolds.

### 3.6 Cell imaging

**Figure 7:**
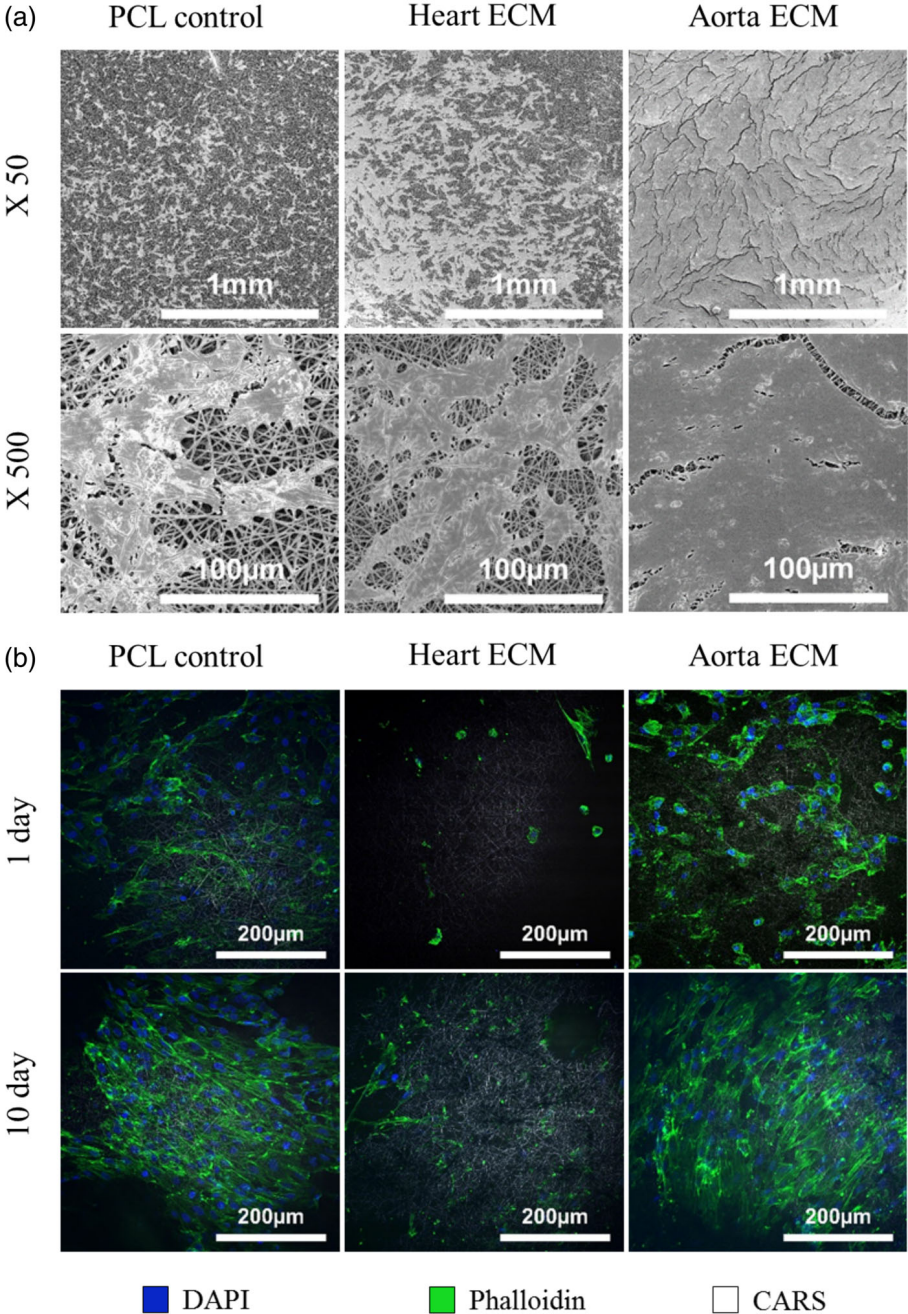
(a) Representative SEM images of scaffolds and a functional layer of HUVEC cells. The aorta ECM scaffold showed 100% confluence after 10 days of culture compared with approximately 30% for the PCL scaffold and 50% for the heart ECM scaffold. (b) Representative coherent antistoke Raman scattering (CARS) images of HUVECs on all three scaffolds at 1 day and 10 days. DAPI staining (blue) highlights the cells nucleus. Phalloidin staining (green) highlights the actin filaments which are predominantly found in the cell cytoplasm. PCL scaffold fibers (white). ECM, extracellular matrix; HUVEC, human umbilical vein endothelial cell; SEM, scanning electron microscope; PCL, polycaprolactone

Representative SEM images taken using a Hitachi S4700 fuelled emission SEM (Hitachi) show the functional cell layers on scaffolds after 10 days of culture (Figure 7a). Interestingly, the aorta ECM scaffold appears to be 100% confluent with HUVECs, compared with approximately 30% for the PCL scaffold and 50% for the heart ECM scaffold. These results validate the cell viability and DNA quantification results, suggesting that the incorporation of aorta ECM into the scaffold improved HUVEC proliferation. Furthermore, all three scaffolds show similar HUVEC morphology.

Representative images taken using coherent antistoke Raman scattering further confirm that the incorporation of aorta ECM into the scaffold had a favorable effect on HUVECs (Figure 7b). After 1 day of growth, the cells on the aorta ECM scaffold already appear greater in number and have a higher quantity of visible actin filaments. The heart ECM scaffolds show very poor cell attachment after 1 day, which matches the cell viability and DNA quantification results. Furthermore, cell number appears to increase for all scaffolds between day 1 and day 10. Interestingly, the actin filaments appear more characterized after 10 days, which is a sign of maturing cells (Huber et al., 2013).

### 3.7 Gene analysis

**Figure 8:**
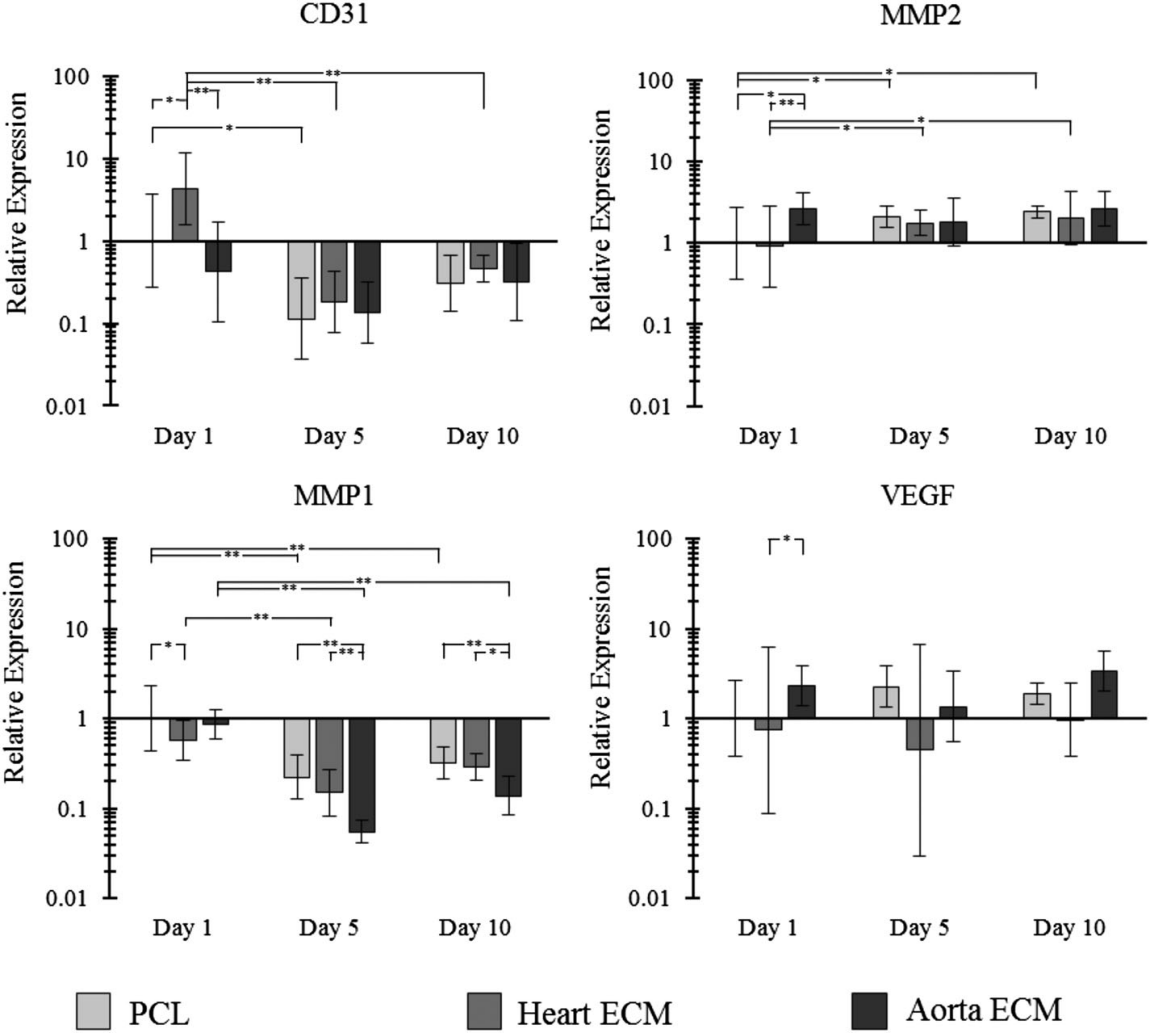
Real time qPCR data using the 2^−ΔΔ*Ct*^ method showing the expression of four genes (MMP1, MMP2, CD31, and VEGF) relative to GAPDH. All results are normalized to the PCL scaffold on day 1. *N* = 5, data shown as mean ± 1 *SD*, one‐way ANOVA with Fishers' post hoc performed (*p < .05 and **p < .01). ANOVA, analysis of variance; CD31, cluster of differentiation; GAPDH, glyceraldehyde 3‐phosphate dehydrogenase; MMP1, matrix metalloproteinase‐1; MMP2, matrix metalloproteinase‐2; PCL, polycaprolactone

Multiple genes associated with vascular function were assayed for their expression, including cluster of differentiation (CD31), matrix metalloproteinase‐1 (MMP1), matrix metalloproteinase‐2 (MMP2), and vascular endothelial growth factor (VEGF) (Figure 8). Statistical significance was noted in CD31 expression changes, with downregulation observed for all three scaffolds between day 1 and day 5. Within the day 1 scaffolds, the heart ECM scaffold showed higher CD31 expression than the two other scaffolds. MMP1 expression showed downregulation over time for all three scaffolds, with the biggest reduction in expression seen for the aorta ECM scaffold. Significantly lower expressions were also noted for the aorta ECM scaffolds at 5 and 10 days when compared with the two other scaffolds. Conversely, MMP2 expression was seen to increase over time, with significant increases for the PCL and heart ECM scaffolds. Furthermore, the aorta ECM scaffold had a significantly higher expression at day 1 compared with the PCL and heart ECM scaffolds. VEGF showed no real trend over time, with the only exception being that aorta ECM scaffold had a significantly higher expression than the heart ECM scaffold at day 1.

## 4 DISCUSSION

Polymer scaffolds have long been utilized in tissue engineering as a platform for tissue regeneration (Dhandayuthapani, Yoshida, Maekawa, & Kumar, 2011; O'Brien, 2011; Saha, Pollock, Schaffer, & Healy, 2007). Physical characteristics can be designed into the scaffold to support cellular activity and promote differentiation, proliferation, and deposition of new functional ECM (O'Brien, 2011). In this study, we have used PCL as the polymer component as it is commonly used in tissue engineering due to its physical and chemical properties, especially in electrospinning where it has been used in a variety of tissue engineering applications (Burton et al., 2017; Dunphy, Reid, Burton, et al., 2018; Gao et al., 2017; Grant et al., 2017; Sundaramurthi et al., 2014). Alongside polymers, decellularized tissue has been used in tissue engineering due to its cell‐supporting physical and chemical characteristics (Lu et al., 2013; Tapias & Ott, 2014). The aim when decellularizing is to maintain the physical and chemical characteristics of the remnant ECM (Gilbert et al., 2006). In this study, we used SDS (at a concentration of 0.5%), which is a commonly used ionic detergent for decellularizing tissues (Nakayama, Batchelder, Lee, & Tarantal, 2010; Oberwallner et al., 2014; Ott et al., 2008; Ott et al., 2010; Ross, Williams, & Batich, 2009) and successfully reduced DNA content approximately 8–14 fold across the two tissue types (Figure 3a). It is a very effective means of removing nuclear components and cytoplasmic proteins (Woods & Gratzer, 2005) and has been used with varying concentrations ranging from 0.1 to 4%, with results showing complete removal of cellular material while maintaining structural integrity and a network of laminin and collagen (Nakayama et al., 2010; Oberwallner et al., 2014; Ott et al., 2008; Ott et al., 2010; Ross et al., 2009). H&E staining confirmed the removal of the DNA content from the two tissue types after decellularization, and the Picrosirius red staining confirmed that the collagenous structure of the ECM was maintained during decellularization (Figure 3b). The FTIR results for the two ECMs showed peaks in the areas associated with structural proteins such as collagen and elastin, suggesting that the decellularizing process maintained the protein's integrity (Cheheltani, McGoverin, Rao, et al., 2014).

As previously described, polymers and ECM both have their advantages when used as scaffolds for tissue engineering (Dhandayuthapani et al., 2011; Tapias & Ott, 2014). In this study, we have combined the two in an attempt to harness the beneficial characteristics from each to create a bioscaffold more suited for vascular tissue engineering. Recently, work has focused on combining the two to take advantage of these characteristics, in a similar vein to this study (Gao et al., 2017; Hong et al., 2008; Jahnavi et al., 2015; Jiang et al., 2015; Wu et al., 2007). For example, coating vascular ECM with polymers has been implemented before, with the aim of improving biochemical and mechanical performance (Jahnavi et al., 2015; Wu et al., 2007). These studies both noted improved mechanical properties. However, the final structure of the scaffold is limited by the size and architecture of the ECM. By integrating the ECM directly into the polymer fibers, as we have done in this study using electrospinning, we are able to create a highly tailored and physically repeatable structure that both includes the biochemical cues found in native ECM, and has the mechanical strength of the electrospun polymer. Moreover, as found in this study and previous, there are mechanical and biochemical benefits to combining ECM with polymers (Jahnavi et al., 2015; Wu et al., 2007).

The inclusion of ECM into the PCL fibers was confirmed through the use of FTIR. The two PCL/ECM scaffolds shared absorbance peaks with the PCL only scaffold and the ECM alone. These peaks were noted at 1720, 1654, and 1,541 cm^−1^, and have assignments with the carbonyl group in PCL, and the amide I and amide II groups found in ECM (Camacho et al., 2001; Elzein et al., 2004). The changes in absorbance found at these peaks due to the addition of the two ECMs were quantified, as shown in Tables 2 and 3, and were found to be in line with what we would expect from literature (Gao et al., 2017).

The technique proposed in this study solubilizes vascular ECMs into a PCL/HFIP solution; a method previously used with decellularized meniscus and cartilage ECM (Gao et al., 2017; Garrigues et al., 2014). Gao et al. used a range of PCL and meniscus ECM concentrations up to 8 and 6% w/v, respectively. While this ECM concentration is substantially higher than the ones used in this study, they found that the addition of ECM had a positive effect in reducing contact angle, a phenomena also noted with the inclusion of aorta ECM in this study. Similarly, Garrigues et al. dissolved decellularized cartilage ECM into a solution of PCL and HFIP at 8% w/v, showing that ECM could successfully be incorporated into an electrospun polymer fiber and provide a platform for cell growth (Garrigues et al., 2014). Gene expression changes were observed with the addition of cartilage ECM, in similar vein to this study. We have shown that the same procedure can be used with vascular tissues, with the incorporation of ECM altering mechanical and cellular performance.

The inclusion of ECM into the electrospun polymer fibers had the effect of increasing the scaffold's tensile Young's modulus, as noted in Table 4. Significant differences were seen at the 0–1% strain band for both scaffolds where the heart ECM was 23.2% higher and aorta ECM 28.4% higher, and the 1–2% strain band for aorta ECM which was 31.0% higher. This can be explained by these native vascular ECMs being made up of constituent proteins such as collagen, which has been found to have much higher stiffnesses at lower strains than PCL alone (bulk properties), 1–21 GPa for collagen compared with 300–400 MPa for PCL (Eshraghi & Das, 2010; Wenger, Bozec, Horton, & Mesquida, 2007). Interestingly, this phenomenon has also been noted when electrospinning combinations of liver ECM, fibronectin, and collagen with polylactic acid (Grant, Hallett, Forbes, et al., 2019). They found that the scaffold's Young's modulus increased by between 13 and 66% depending on which protein was blended (Grant et al., 2019).

A trend of decreasing MMP1 expression for all three scaffolds was noted. MMP1 is an enzyme involved in the breakdown and remodeling of collagen (one of the major components of vascular ECMs) (Batra et al., 2012). Its downregulation suggests that the cells are not trying to breakdown the collagen within the ECM, suggesting that they are in a position of homeostatic equilibrium with their scaffold. A significant downregulation was noted across all three scaffolds between day 1 and day 5, with no real change between day 5 and day 10. Interestingly, the largest downregulation was noted in the aorta ECM scaffold. While this downregulation may suggest that there is a change in collagen content within the ECM scaffolds, similar trends were noted between the ECM scaffolds and the PCL only scaffold suggesting that scaffold architecture is likely the biggest driver in gene expression and that the ECM is having a limited effect on gene activity.

In contrast, a significant upregulation of MMP2 was noted in all three scaffolds after 5 and 10 days. Furthermore, after 1 day, a significantly higher expression was seen in the aorta ECM scaffold compared with the two other scaffolds. This enzyme is involved in the breakdown and remodeling of gelatin (another major component of vascular ECMs) (Wiseman et al., 2003). Its upregulation suggests the presence of gelatin in the ECM scaffolds. We noted an upregulation across both ECM scaffolds, as well as the PCL only scaffold after 10 days of culture, with no significant differences noted between the three scaffolds. This suggests that this upregulation is unlikely to be caused by the presence of gelatin in the ECM scaffolds and is probably a result of something other than the constituents of the two native ECMs, such as scaffold architecture, as previously mentioned.

On the other hand, no real trend in VEGF expression was noted. VEGF is a signal protein that stimulates the formation of new blood vessels (angiogenesis) in tissue regeneration (Ferrara, Gerber, & LeCouter, 2003). Its overexpression is also associated with tumor growth and intraocular neovascular disorders (Ferrara et al., 2003). During cellular proliferation, an upregulation of VEGF would be expected (Nayak & Brenner, 2002). It has been suggested that HUVECs “overexpress” VEGF in the first 24 hr, which may explain this down regulation over time compared with the day 1 scaffolds (Imaizumi et al., 2000).

A downward trend in CD31 expression was seen for all three scaffolds. CD31, also known as PECAM1 (platelet endothelial cell adhesion molecule 1) is a protein found on the surface of many cells and is involved in angiogenesis and integrin activation. Overexpression of CD31 can inhibit morphogenesis in cells (Sheibani & Frazier, 1998). Its downregulation suggests that: the HUVECs are struggling to adhere to each other (Baldwin, Shen, Yan, et al., 1994), which SEM and fluorescence microscopy images disprove, or the HUVECs are not attempting to create new vasculature (angiogenesis) (Wang et al., 2016). This is the most likely reason that can be attributed to the issue of poor cellular infiltration which is required in order to achieve full tissue regeneration. A lack of infiltration in this study resulted in a monolayer of cells growing on the surface of the scaffold. Studies have shown the contribution of porosity and pore interconnectivity on cell survival and HUVEC angiogenesis (Munir & Callanan, 2018; Xiao, Wang, Liu, et al., 2015). Despite this, the ECM scaffolds in this study are still viable platforms for cell attachment, and cellular infiltration can be improved by either increasing the mean pore diameter (Murphy, Haugh, & O'Brien, 2010; Phipps, Clem, Grunda, Clines, & Bellis, 2012; Wu & Hong, 2016), or by increasing the hydrophilicity of the scaffold through plasma treatment and the addition of functional groups (Burton et al., 2017; Dettin et al., 2015), two easily applicable techniques.

In this study, there was a noticeable trend in the data suggesting that the aorta ECM scaffold had enhanced adhesive properties compared with the two other scaffolds. DNA quantification and cell viability were both higher for the aorta ECM scaffold at 24 hr. The FTIR results showed a greater increase in absorbance at the amide I and amide II peaks when aorta ECM was added to the fiber compared with when heart ECM was added to the fiber. FTIR is a surface analysis method that has been shown to measure to depths ranging from 0.2 to 5 μm (Kazarian & Chan, 2013). The fibers in this study are approximately 1 μm thick suggesting that the shifts in absorbance may be due to the location of the ECM within the fiber. Higher absorbance in the aorta ECM scaffolds may be caused by the ECM mobilizing onto the surface of the fibers, where they would be more readily picked up by FTIR. Furthermore, the aorta ECM scaffold was found to have a lower contact angle than the two other scaffolds, which has been shown to result in higher cell adhesion and proliferation (Dowling, Miller, Ardhaoui, & Gallagher, 2011; Gao et al., 2017). The differences in contact angle may be due to the components of ECM at the fiber surface increasing the surface energy of the scaffold (Faucheux, Schweiss, Lützow, Werner, & Groth, 2004).

There are some limitations to this work. First, a thorough investigation of electrospinning in HFIP's denaturing effect on the ECM is required. Previous studies have shown that HFIP can have a denaturing effect on various proteins, including collagen (Agarwal, Wendorff, & Greiner, 2008; Herskovits, Gadegbeku, & Jaillet, 1970; Zeugolis et al., 2008), with one study suggesting that approximately 45% of the triple helical structure of collagen became denatured (and becoming gelatin) when dissolved in HFIP (Yang et al., 2008), which would suggest that the ECM used in this study could be denatured. They also found that pure collagen fibers were water soluble when they were not crosslinked, suggesting that crosslinking would increase the integrity of electrospun protein fibers (Yang et al., 2008). However, it has also been shown that crosslinking electrospun ECM protein scaffolds drastically alters their morphology, often leading to large reductions in porosity (Heydarkhan‐Hagvall et al., 2008), which has been shown to negatively impact certain cells (Badylak & Gilbert, 2008; Lowery, Datta, & Rutledge, 2010). Furthermore, PCL/gelatin blended scaffolds have shown loss of protein when cultured in both PBS and cell culture medium (Marrese, Cirillo, Guarino, et al., 2018). They found that surface roughness increased over a culture period of 9 days due to the gelatin dissolving from the scaffold and interestingly found that this was more pronounced in culture medium than in PBS (Marrese et al., 2018). Likewise, collagen/elastin/polymer (elastin and collagen being the two most prominent ECM proteins) blends with approximately 75% crosslinking have shown varying degrees of mechanical integrity when in culture conditions. Over the course of 8 weeks of culture, reductions in inner diameter ranging from 10% (PCL and PLLA) to 70% (PLCL) were seen depending on the polymer used. This reduction in mechanical integrity could potentially be explained by the dissolution of collagen and elastin from the scaffold fibers. In addition to this, the electric field which the ECM particles are subjected to may also have a denaturing effect. It has been shown that high voltages can alter the structure of proteins (Freedman, Haq, Edel, et al., 2013). Therefore, while both collagen denaturing and protein dissolution are possible during processing, the retained bioactivity from the ECM within the scaffold outweighs the losses seen (Badylak, Freytes, & Gilbert, 2009; Choudhury, Tun, Wang, & Naing, 2018).

SDS has been shown to induce protein denaturing and requires further study on its effects with vascular ECMs (Bhuyan, 2010). While this study did show the successful maintenance of collagen in both tissues after decellularization (Picrosirius Red staining in Figure 3b), other ECM proteins such as elastin were not looked at. However, studies have shown successful decellularization using SDS without damaging the components of the ECM (Garrigues et al., 2014; He & Callanan, 2013; Oberwallner et al., 2014; Sullivan et al., 2012). Furthermore, a thorough proteomic study of the ECM components and the cellular secretome would act as validation for the PCR results and would give a better understanding of which proteins lead to the improved adhesive properties (Chang, Dalgliesh, Lopez, et al., 2016; Hathout, 2007). Further analysis on how the ECM was incorporated into the electrospun fibers is required. Studies have shown contradictory results with some showing complete protein integration into the fibers (Gao et al., 2017; Garrigues et al., 2014), and others showing globular protein microparticles in the fibers (Chakrapani, Gnanamani, Giridev, Madhusoothanan, & Sekaran, 2012). However, the studies which incorporated ECM into electrospun fibers, along with this study, showed improvements in cellular and mechanical performance, suggesting that there is a large potential for these hybrid bioscaffolds in tissue engineering.

## 5 CONCLUSION

In this study, we have shown that the integration of decellularized vascular ECM into an electrospun polymer fibers has promise for vascular tissue engineering applications. We successfully combined decellularized ECM from bovine myocardium and aorta into a PCL/HFIP solution and electrospun it into a network of fibers. Our results show that the inclusion of these ECMs had both effects on the performance of seeded HUVECs and on the mechanical characteristics of the scaffold. Cell viability was shown to increase with the inclusion of aorta ECM in the scaffold. Furthermore, Young's modulus was increased through the addition of both vascular ECMs, and hydrophilicity was increased with the addition of aorta ECM. The inclusion of aorta ECM resulted in a stiffer scaffold that had improved biomechanical properties for the attachment of cells—leading to the increased cell adherence noted in this study. The method we have described combines the controllable physical properties of the polymer (PCL) with the biochemical and mechanical properties of the ECM (aorta and myocardium wall) to create a tailored hybrid bioscaffold. We believe that the combination of a natural ECM and a synthetic polymer to generate a hybrid bioscaffold has many potential applications in vascular tissue engineering.

## Supporting information


**Figure S1** FTIR absorbance spectra of all 5 samples for each group for **A)** aorta ECM scaffolds and **B)** heart ECM scaffolds.
